# Can dexmedetomidine protect against surgical stress response?

**DOI:** 10.1002/ctm2.96

**Published:** 2020-06-28

**Authors:** Junichi Saito, Daqing Ma

**Affiliations:** ^1^ Division of Anaesthetics Pain Medicine and Intensive Care Department of Surgery and Cancer Faculty of Medicine Imperial College London London UK; ^2^ Department of Anesthesiology Hirosaki University Graduate School of Medicine Hirosaki Japan

Surgical stress, pain, and complications are associated with postoperative morbidity and mortality.^1^ Various strategies have been implemented to reduce the stress response,^2^ for which anesthetics/techniques in attenuating perioperative stress and improving outcomes have been reported in several clinical settings.^2^ Recently, the effectiveness of dexmedetomidine on perioperative stress response as well as on short‐ and long‐term outcomes have been well documented.^3^, ^4^ In the current issue, Zhang and his co‐workers^5^ reported a randomized controlled trial (RCT) investigating the effects of dexmedetomidine against perioperative stress response during and after thoracic surgery. Total 135 patients underwent lung surgery were randomly divided into three groups, general anesthesia alone, general anesthesia combined with thoracic paravertebral block (TPVB) mixed with dexmedetomidine, and placebo. They found that, when compared to TPVB with 0.5% ropivacaine alone, TPVB in combination with dexmedetomidine significantly decreased the inflammatory mediators of IL‐6 and TNF‐α, decreased apoptosis index and lung injury score of lung tissue, and promoted favorable changes of various proteins (HIF‐1a, Tom20, Bcl‐2, and others) in an additive or synergistic manner. Additionally, they also found that, when compared to general anesthesia alone, TPVB or thoracic epidural anesthesia maintained better immune function.

Several strengths of this study were noticed: (1) multimeasurements and time points and (2) direct evidence of biomarkers and very subjective assessments. The magnitude of surgical stress depends on surgery type. Unlike minor surgery, major surgery in general triggers high levels of stress. However, this “old” concept may not be right because this study was demonstrated that the thoracoscopic surgery caused very measurable traumatic stress response. This study clearly demonstrated that dexmedetomidine, even single injection but not continuous infusion, had various preferable effects against perioperative stress response, which was failed in the previous studies.^6^ An additional strength of this study is that the samples obtained from patients were analyzed and those data further supported other clinical end points.

However, this study is not without limitations. First, the impact of dexmedetomidine on the prognosis of patients, for example morbidity and mortality, was not assessed in the study. Second, even though anti‐inflammatory effect of dexmedetomidine was clearly showed, the present study failed to show the benefits of the short‐term clinical outcomes including acute pain core and incidence of chronic pain, though there were several benefits compared with general anesthesia alone. This might be due to inadequate sample size (only 41 patients received dexmedetomidine) because the sample size was based on the laboratory data not clinical outcomes. Third, the adverse event of dexmedetomidine, especially hypotension, bradycardia, and other cardiovascular events were not reported, although, compared with intravenous injection, its paravertebral injection might be less notified. The evaluation of those adverse events are crucial because previous large RCTs revealed that perioperative hypotension was an independent predictor of myocardial infarction^7^ and stroke and death,^8^ which was closely associated with the beta‐blocker and a_2_‐adrenoreceptor agonist (clonidine).

A recent meta‐analysis^6^ reported that perioperative use of dexmedetomidine effectively attenuated stress response; blood epinephrine, norepinephrine, cortisol, and blood glucose were significantly decreased by dexmedetomidine in surgical patients. Dexmedetomidine use also decreased the pro‐inflammatory cytokines such as IL‐6, TNF‐α, and C‐reactive protein and increased NK cells, B cells, and CD4+ cells, and the ratio of CD4+:CD8+ and Th1:Th2. These preferable effects of dexmedetomidine in surgical patients were likely due to its attenuation the hypothalamic‐pituitary‐adrenal axis and sympathoadrenal “tone.” Excess stress response is associated with poor postoperative outcomes including the high incidence of postoperative delirium, postoperative cognitive dysfunction,^9^ myocardial infarction, and death.^10^ Indeed, Su and colleagues^3^ conducted an RCT to evaluate the incidence of postoperative delirium. They included 700 elder patients after noncardiac surgery and divided the patients into two groups: either placebo (n = 350) or dexmedetomidine (n = 350), and revealed that the incidence of postoperative delirium was significantly lower in the dexmedetomidine group (9%) compared with the placebo group (23%; odds ratio 0.35, 95% confidence interval (CI) 0.22‐0.54). Kawagoe et al^4^ also conducted multicenter RCT to evaluate the impact of dexmedetomidine on clinical outcome of sepsis patients with mechanical ventilation. They included 201 sepsis patients and divided the patients into two groups: receiving either sedation with dexmedetomidine (n  =  100) or sedation without dexmedetomidine (n  =  101). They showed that 28 days mortality was tend to be low in the dexmedetomidine group (22.8%) compared with the control group (30.8%; hazard ratio, 0.69; 95% CI, 0.38‐1.22; *P*  =  .20) even though due to underpower of this study the difference was not significant. These results suggest that anti‐inflammatory effect of dexmedetomidine has a potential to improve surgical patients’ outcomes, but to date, there is not enough evidence regarding the association between dexmedetomidine and long‐term outcomes. Further RCTs with large sample size are needed to determine the association of dexmedetomidine and long‐term outcomes of surgical patients.

## AUTHOR CONTRIBUTIONS

Junichi Saito drafted the manuscript. Daqing Ma conceived the study idea and is the archival author. All authors participated to write the manuscript.

Reprints will not be available from the authors.

## CONFLICT OF INTEREST

All authors declare no conflict of interest.
